# Correction Schemes for Absolute Binding Free Energies Involving Lipid Bilayers

**DOI:** 10.1021/acs.jctc.1c01251

**Published:** 2022-03-22

**Authors:** Zhiyi Wu, Philip C. Biggin

**Affiliations:** Department of Biochemistry, South Parks Road, Oxford OX1 3QU, U.K.

## Abstract

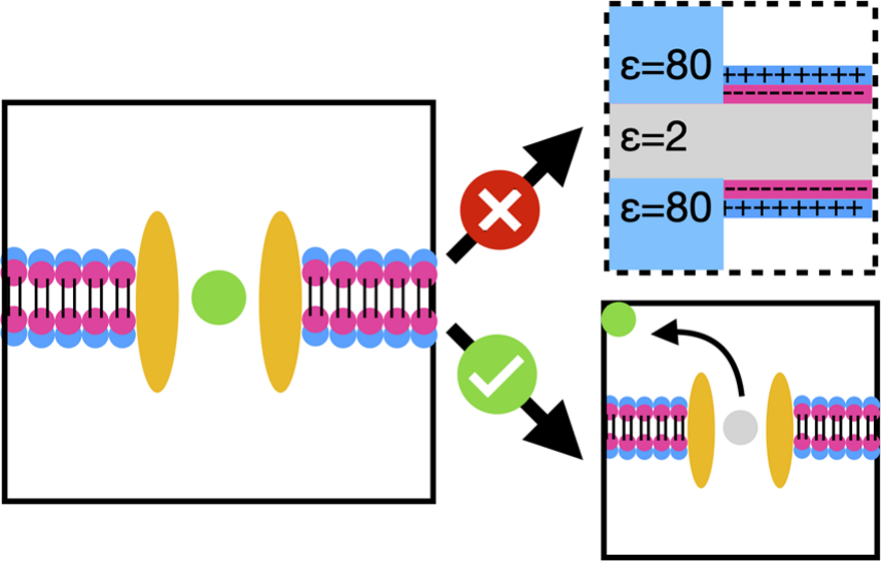

Absolute binding free-energy (ABFE) calculations are playing an increasing role in drug design, especially as they can be performed on a range of disparate compounds and direct comparisons between them can be made. It is, however, especially important to ensure that they are as accurate as possible, as unlike relative binding free-energy (RBFE) calculations, one does not benefit as much from a cancellation of errors during the calculations. In most modern implementations of ABFE calculations, a particle mesh Ewald scheme is typically used to treat the electrostatic contribution to the free energy. A central requirement of such schemes is that the box preserves neutrality throughout the calculation. There are many ways to deal with this problem that have been discussed over the years ranging from a neutralizing plasma with a post hoc correction term through to a simple co-alchemical ion within the same box. The post hoc correction approach is the most widespread. However, the vast majority of these studies have been applied to a soluble protein in a homogeneous solvent (water or salt solution). In this work, we explore which of the more common approaches would be the most suitable for a simulation box with a lipid bilayer within it. We further develop the idea of the so-called Rocklin correction for lipid-bilayer systems and show how such a correction could work. However, we also show that it will be difficult to make this generalizable in a practical way and thus we conclude that the use of a “co-alchemical ion” is the most useful approach for simulations involving lipid membrane systems.

## Introduction

The accurate calculation of the free energy (FE) of many processes such as ligand binding,^1^ change of protonation state,^2^ or the influence of mutations^3^ is a major focus of modern computational biochemistry.^4^ The current state-of-art approach is to construct a periodic computational box where the protein is solvated in explicit water molecules described using a molecular mechanics (MM) force field and to perform alchemical transformation or path sampling to obtain the desired properties. The long-range electrostatics are usually calculated with lattice-sum methods like particle mesh Ewald (PME).^5^ However, PME demands the simulation box to be neutral. Thus, for the annihilation/decoupling of charged ligands during binding free-energy calculations, protonation free-energy calculations, or protein mutation calculations where the net charge of the simulation box is perturbed, this has to be dealt with in some way and should not be ignored.^6^

Various solutions to this problem have been suggested. The most extensively employed strategy is where a neutralizing plasma is evenly distributed throughout the simulation box to ensure the overall neutrality is maintained. Though such a plasma does indeed maintain the neutrality of the simulation box, it generates a size-dependent artifact. This size-dependent artifact exists in free-energy estimates of ligand binding free-energy calculations involving changes of charge,^7^ charge-perturbing protein mutations,^8^ and protonation free-energy calculations.^9^

To tackle this finite-size effect, many approaches can be taken, ranging from ignoring charge-changing mutations,^10^ calculating an explicit correction^11^ through to incorporating a co-alchemical ion to counteract directly the charge effect.^12^

PME is used widely to compute long-range electrostatics, and the finite-size effect has been well characterized in such calculation systems (see Rocklin et al.^11^ and references therein). Thus, one solution would be to avoid using PME. A possible alternative in this regard is the reaction field (RF) method^13^ or modified RF methods, where the cutoff is based on charge groups instead of atoms^14^ or the anisotropic RF method.^15^ All of these methods have thus far been shown to provide comparable performance to PME without the finite-size effect. Though RF methods might work well for homogeneous systems, such as a protein in a pure water solvent, care must be taken for inhomogeneous conditions such as a lipid–water system where the lipid hydrophobic core has a very different dielectric constant compared with bulk water. In such instances, PME may well be a better choice.^16^

Another alternative approach is to use force-switching electrostatics, which has been shown to perform better than PME in terms of dealing with a charged simulation box.^17^ For some special cases, such as computing the binding energy of ligand, pure quantum mechanics (QM)^18^ or QM/MM^19^ Hamiltonians are also considered as ways to avoid the size-dependent artifact. On the other hand, electrostatic interactions computed with Poisson–Boltzmann (PB) or generalized-Born (GB), where nonperiodic bound conditions are assumed, can also give results free from finite-size effects.^20^

For ligand binding free-energy calculations, one can obtain the free energy of moving the ligand from the protein to the solvent via double-decoupling/annihilation methods, where the free energy of decoupling/annihilating the ligand from the protein and water is computed separately and the difference is the binding free energy. Thus, when the ligand is charged, the decoupling/annihilation of the ligand will remove charge from the simulation system and give rise to a finite-size effect. One solution for this specific problem would be physically moving the ligand out of the protein into the water phase. Since the ligand is always in the same box during this transition, the charge of the box stayed neutral. The free energy of binding can then be recovered by path-sampling techniques^21^ such as the attach–pull–release scheme.^22^

When path-sampling techniques are used to compute the ligand binding free energy, the starting point of the simulation is the ligand bound to the protein and the end point is the ligand in the bulk solvent, quite remote from the protein. To compute the free energy, the whole physical path needs to be sampled. Thus, the calculations are very expensive, especially if the path is long. One solution is to preserve the start and end points and bridge them with alchemical transformations. For ligand binding free-energy calculations, the ligand is decoupled/annihilated in the protein and is coupled/created in the solvent phase at the same time to keep the box neutral.^23^

This approach has been generalized to other types of calculations and is sometimes also referred to as the double-system/single-box approach,^24,25^ where the whole cycle of the alchemical transformations is done in the same box so that the charge is always conserved. The approach has been used to understand the effect of protein mutation on the stability of the protein, where the original residue in the protein is mutated to the target residue and the target residue in a tripeptide (to mimic the unfolded protein) is mutated back to the original residue.^3^ A similar approach has been used to investigate the effect of protein mutation on the stability of a protein dimer, where the original residue in the dimer is mutated to the target residue and the target residue in the monomer protein is mutated back to the original residue.^26^ Although this approach is effective, it requires a much larger simulation box, which is not computationally efficient.

Perhaps, the simplest approach designed to account for the change of net charge during an alchemical transformation is to employ an additional “co-alchemical ion”, which changes its charge at the same time as the main perturbation such as to keep the box neutral overall. The simplicity of the implementation of the co-alchemical ion makes it the ideal solution to automatic workflows, which are being increasingly developed and employed.^27,28^

Overall, the strategy that has been most commonly adopted has been the neutralizing plasma approach that employs a postsimulation correction scheme. The simplest scheme is a correction derived for a naked point charge in a continuum medium.^29^ However, given that for modern simulations, solvents are usually represented as discrete molecules and the protein systems are too complex to be represented as a continuum, Rocklin expanded the scheme and used adaptive Poisson–Boltzmann solver (APBS) calculations to account for the difference between the protein and a continuum medium.^11^ This extended scheme, commonly referred to as the “Rocklin correction”, has been used in many studies,^24,30^ and its accuracy has been verified by other groups.^31^ For soluble proteins, the co-alchemical ion approach and the Rocklin correction give similar results.^32^ The finite-size effect has also been seen in simulations using polarizable force fields (AMOEBA),^33^ and the Rocklin correction has been shown to be able to correct for that.^34^ Similarly, electrostatic interactions computed using multipole methods exhibit finite-size effects and these can also be corrected with the Rocklin correction.^35^ Given the complexity of the correction, a simplified version is sometimes used in automatic workflows.^36^

However, nearly all calculations to date have been performed with soluble proteins and it remains unclear as to how well these corrections can be applied to membrane protein systems. It has already been shown that the neutralizing plasma can affect the interpretation of membrane systems.^37^ Though the Rocklin correction has been shown to improve the result of membrane systems,^38^ the original derivation does not consider nonwater solvent and it is unclear as to how to incorporate components like lipid membranes. In other simulations where the bulk solvent is not water, path sampling has been used to avoid finite-size effects.^39^

In this study, we explore which of the various methods outlined above would offer the best performance for membrane simulation boxes. As part of this process, we derived a new postsimulation correction scheme, similar to the original Rocklin scheme. However, our results suggest that the co-alchemical ion approach may be the preferred route forward.

## Results

### Rocklin Correction in the Case of a Single Ion in Water

To solve the finite-size artifact during charge-changing free-energy calculations, Rocklin derived a semianalytic correction scheme. The correction converts a periodic boundary condition (PBC) system with box length L (PBC(L)) into a nonperiodic system of infinite size (e.g., a macroscopic droplet) (Figure 1A) so as to remove the size dependency of the system. The correction can be decomposed into two components. The first is the interactions between the system of interest and its periodic neighbors. In periodic systems, when there is a net charge associated with the simulation box, the net charge will be exerting significant electrostatic interactions to the solute in neighboring boxes (Figure 1B) that are not present in the nonperiodic system. The second component is that the solvent will interact with the net charge differently in the nonperiodic system compared with that in the periodic system. In a nonperiodic system, the electrostatic potential (ESP) generated by a point charge would only vanish at an infinite distance. Thus, all of the solvent molecules will reorient in response to this electrostatic potential (Figure 1C). In periodic systems, on the other hand, the electrostatic potential would be set to zero at the boundary due to the presence of the periodic neighbor and the solvent will have different orientations.

**Figure 1:**
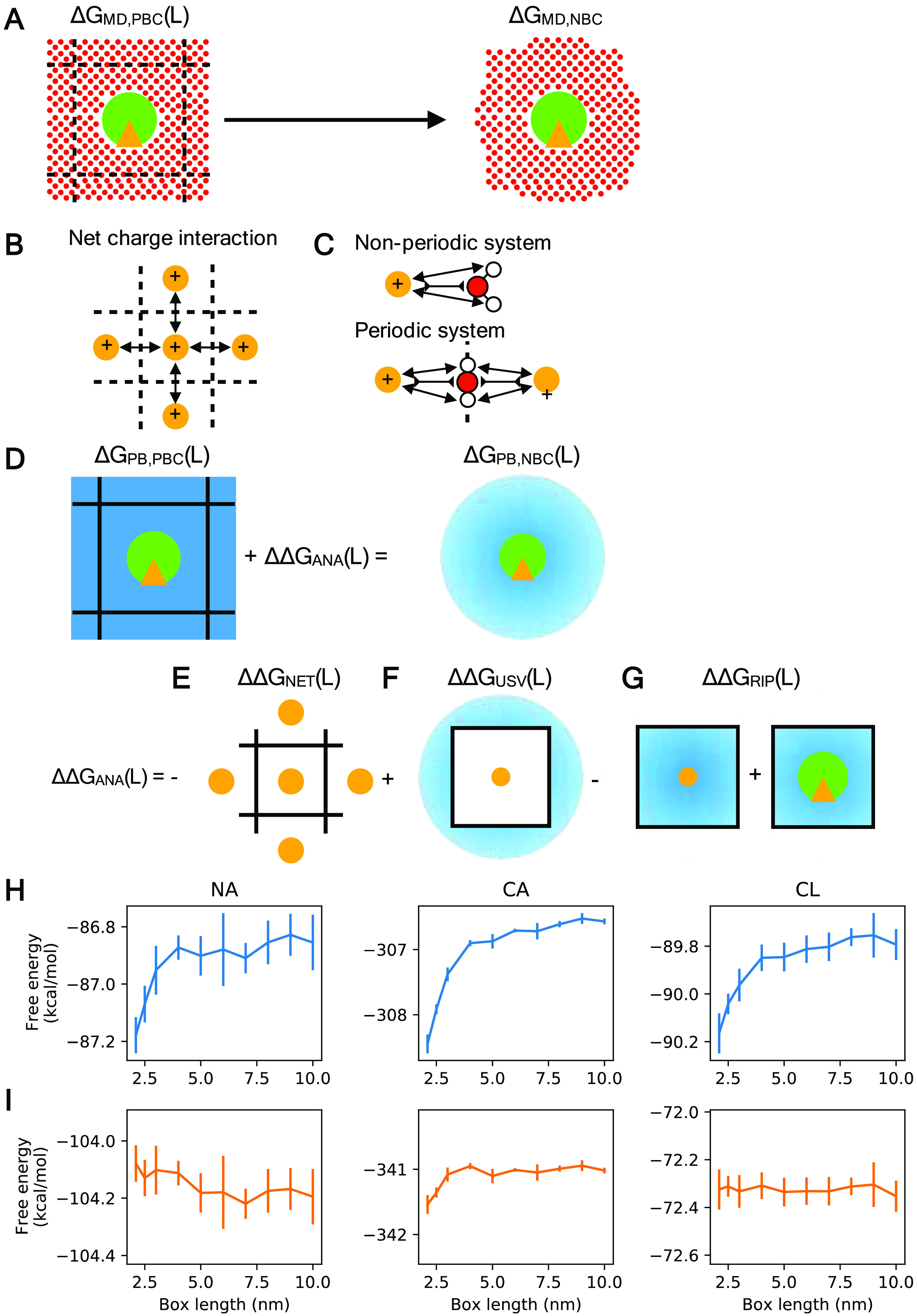
Rocklin correction for a single ion in water. (A) The Rocklin correction defines a semianalytic correction that converts a periodic boundary condition system to a nonperiodic condition system with infinite volume. (B) Under periodic boundary conditions, the solute will interact electrostatically with its periodic neighbor. (C) The presence of the periodic neighbor will affect how the solvent is orientated. (D) After a discrete solvent correction (DSC), a semianalytic correction is used to transform the periodic Poisson–Boltzmann system to a nonperiodic Poisson–Boltzmann system. (E) Periodicity-induced net charge interaction (NET) correction. (F) Periodicity-induced net charge undersolvation (USV) correction. (G) Residual integrated potential (RIP) correction. (H) The uncorrected charge annihilation free energy of the ions Na^+^, Cl^-^, and Ca^2+^ displays size dependency. (I) The same size dependency is eliminated when the Rocklin correction is applied.

To correct the free energy derived from periodic conditions, Δ*G*_MD,PBC_(*L*), Rocklin proposed two different correction terms as shown in eq 1: the discrete solvent correction (DSC) to correct for the solvent effect ΔΔ*G*_DSC_(*L*) and an analytical (ANA) correction ΔΔ*G*_ANA_(*L*) to correct for net charge solute interactions. Together, these result in the free energy under non-PBC conditions Δ*G*_MD,NBC_
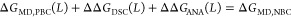
1

During a simulation, the instantaneous solvent rearrangements can give rise to large energy fluctuations. Thus, to obtain the ensemble energy of the system, a large number of frames need to be taken into account. The discrete solvent correction transforms the system from having explicit water molecules into a continuum-electrostatics Poisson–Boltzmann (PB) model, which avoids the necessity of applying the correction to multiple frames, as summarized in eq 2
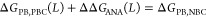
2The analytical correction ΔΔ*G*_ANA_(*L*) then transforms the periodic PB model Δ*G*_PB,PBC_(*L*) into a PB model in a nonperiodic box Δ*G*_PB,NBC_ (Figure 1D). To achieve this transformation, two steps are taken (eq 3): the charge interactions between periodic neighbors are removed by ΔΔ*G*_NET_(*L*) and the polarization effect of the net charge on the medium outside the simulation box can be added by ΔΔ*G*_USV_(*L*)
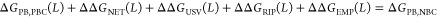
3The ΔΔ*G*_NET_(*L*) is the periodicity-induced net charge interaction (NET) correction, the ΔΔ*G*_USV_(*L*) is the periodicity-induced net charge undersolvation, the ΔΔ*G*_RIP_(*L*) is the residual integrated potential correction, and the ΔΔ_GEMP_(*L*) is an empirical correction.

The leading term of the charge interactions between the periodic neighbors is the net charge interaction, which is corrected with a periodicity-induced net charge interaction (NET) correction ΔΔ*G*_NET_(*L*) (Figure 1E). In ΔΔ*G*_NET_(*L*), the net charge of the system is approximated with a point charge at the center of the simulation box and ΔΔ*G*_NET_(*L*) corrects for the self-interactions between charged species across the periodic boundaries in vacuum.

The polarization effect of the net charge on an infinite medium is corrected for by the periodicity-induced net charge undersolvation term ΔΔ*G*_USV_(*L*) (Figure 1F), which corrects for the solvation of the charged species disrupted by the periodic boundary. Both ΔΔ*G*_NET_(*L*) and ΔΔ*G*_USV_(*L*) are calculated analytically, assuming that the charged species is a naked point charged (naked means no excluded volume) centered in a box of water. The difference between the real system and a point charge is corrected with the residual integrated potential correction (ΔΔ*G*_RIP_(*L*)), which performs a PB calculation to derive the average electrostatic potential difference between the solute and the point charge, where the average electrostatic potential of the point charge is computed analytically (Figure 1G).

To illustrate the effect of the Rocklin correction, we computed the charge annihilation free energy of a single ion in a box of water. The uncorrected charge annihilation free energy exhibits size dependency (Figure 1H), while the corrected free energy does not (Figure 1I).

### Systems with a Lipid Bilayer

As shown above, the Rocklin scheme works very nicely for a box of homogeneous solvent. However, it is currently unclear as to how one should proceed in a nonhomogeneous environment such as a simulation box with a lipid bilayer present. Though many papers have highlighted the specific interactions between the lipid head group and the protein,^40^ lipids are usually included in the simulation to provide the necessary hydrophobic environment to accommodate transmembrane proteins. One could argue that the hydrophobic region would have very little effect on the correction terms that purely deal with electrostatic interactions. Thus, in the first instance, we investigated whether simply ignoring the lipid bilayer would give correct results by investigating a test ion in a lipid–water system to check the accuracy of the correction when the membrane is not taken into account.

Similar to the charge annihilation free energy of a single ion in a box of water, the charge annihilation free energy of a single ion in a lipid–water system exhibits a strong size-dependent effect (Figure 2A). When the Rocklin correction is applied assuming the lipid has no excluded volume and no partial charge, the size-dependent effect is mostly corrected (Figure 2B). However, the corrected free energy still exhibits a small size dependency and is some distance away from the reference charge annihilation free energy (calculated from the charge annihilation free energy of a single ion in water) (Figure 2B). The deviation from the reference free energy is proportional to the net charge of the ion and converges toward 0 with a bigger box size (Figure 2C). Thus, this route, although simple, does not provide a proper route to correcting the electrostatics.

**Figure 2:**
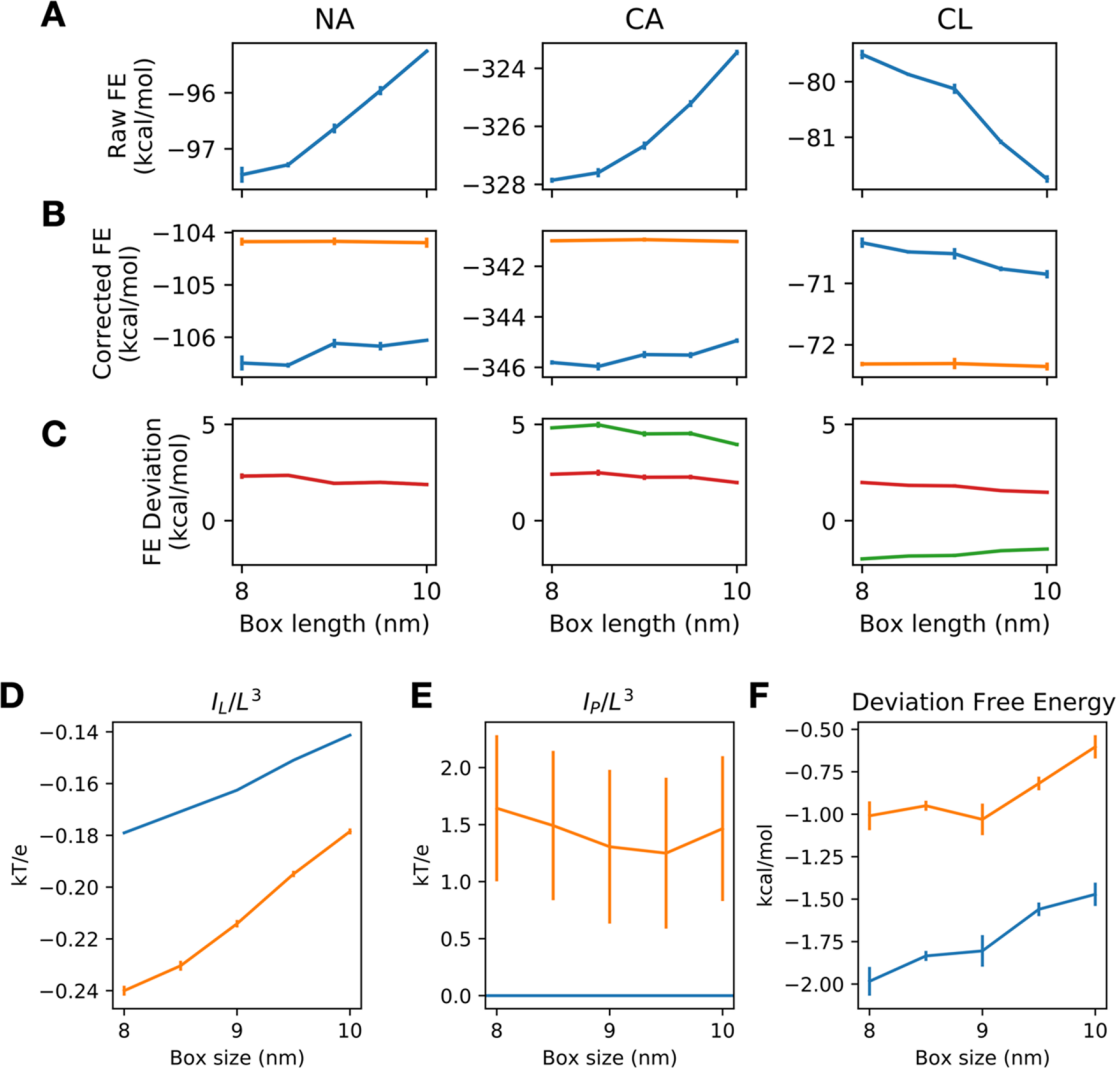
Effect of the Rocklin correction on the annihilation free energy of a single ion in the membrane water system. (A) The uncorrected annihilation free energy (FE) of chloride, sodium, and calcium ion has strong size dependency. (B) The annihilation free energy of a single ion in a lipid membrane with the Rocklin correction applied (blue line) converges toward the reference annihilation free energy of a single ion in pure solvent (orange line). (C) The deviation of the corrected annihilation free energy from the reference free energy (green line), when divided by the charge of the ion, gives a similar profile (red line). (D) The average integrated potential of the ligand in the absence (blue line) and the presence (orange line) of an explicit representation of the lipid. (E) The average integrated potential of the protein in the presence (orange line) and absence (blue line) of the lipid. (F) The deviation from the reference free energy (blue line) shrinks when the lipid is represented explicitly.

### Including the Lipid Bilayer in the Residual Integrated Potential Calculations

Having shown that ignoring the lipid bilayer would give a sizable deviation from the reference value, we tried to incorporate the role of the lipid bilayer into the Rocklin correction. The Rocklin correction corrects the periodicity-induced artifact by deriving an analytical solution (ΔΔ*G*_NET_(*L*), ΔΔ*G*_USV_(*L*)) to correct for the ideal case where the system is represented as a naked charge centered in the box representing the net charge of the system. An additional residual integrated potential ΔΔ*G*_RIP_(*L*) is then used to compute the difference between the real system and the naked charge.

The rationale behind ΔΔ*G*_RIP_(*L*) is that the major difference between a periodic system computed using a lattice-sum method and nonperiodic system computed using Coulombic equations is that in a periodic system, the average electrostatic potential of the simulation box is constrained to zero, whereas such a constraint is not present in nonperiodic systems. Thus, to transform the system to a nonperiodic condition, we need to obtain the energy of charging the system in nonperiodic systems, which is the product of the average electrostatic potential, computed as the integrated electrostatic potential of the simulation box over the box volume (*I*/*L*^3^) and the net charge of the system (*Q*), as shown in eq 4
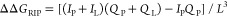
4For the specific case of ligand binding free-energy calculations, the correction is computed as the difference of the charging energy between the apo protein and the protein–ligand complex. The charging energy of the protein–ligand complex is the product of the net charge of the complex (*Q*_P_ + *Q*_L_) and the average electrostatic potential of the complex ((*I*_PL_)/*L*^3^). Rocklin applied an approximation that *I*_PL_ = *I*_P_ + *I*_L_, where *I*_P_ and *I*_L_ are the integrated potential computed using adaptive Poisson–Boltzmann solver (APBS) calculations with both ligand and protein as excluded volumes. The *I*_P_ is computed with protein having partial charges, while *I*_L_ is computed with ligand having partial charges. Thus, the sum of *I*_P_ and *I*_L_ would be *I*_PL_ and *I*_P_ can be reused during the apo protein calculation, which is the product of the average electrostatic potential of the protein (*I*_P_/*L*_3_) and the net charge of the protein (*Q*_P_).

The simplest approach of incorporating the effect of the lipid would be to consider the lipid as part of the protein. Thus, the lipid would form part of the excluded volume in the *I*_L_ and *I*_P_ calculations and contribute electrostatically to the *I*_P_ calculation. If we consider the example of the charging free energy of a single ion in a membrane water system, when the lipid is absent, the *I*_P_ term will be 0. If the lipid is included in the APBS calculation in the same manner as the protein, the lipid contributes to the APBS calculations as individual atoms with a partial charge. These contributions for *I*_L_ and *I*_P_ deviate from the case where lipid is not taken into account (Figure 2D,E). The deviation in *I*_L_/*L*_3_ is very small (∼0.05 kT/e), showing that treating the lipid bilayer as a hydrophobic slab has little impact on the correction. On the other hand, the deviation in *I*_P_/*L*^3^ is sizable, showing that the lipid bilayer drastically changes the electrostatic environment through charge interactions. It is also worth noting that due to the frame-to-frame fluctuation of the lipid, a standard deviation of ∼0.2 kT/e is observed for *I*_P_/*L*^3^, showing that multiple frames are required to obtain an accurate estimate of *I*_P_/*L*^3^. By using the new *I*_P_/*L*^3^ and *I*_L_/*L*^3^ in the ΔΔ*G*_RIP_(*L*) calculations, the deviation from the reference value is reduced (Figure 2F), but there is still considerable residual deviation. However, it is worth noting that this may be an overestimate of bias in the uncorrected result because even ABFE, via the double-decoupling nature, does have some degree of error cancellation.

### Defining the Nonperiodic Condition for Membrane Systems

Though the inclusion of an explicit lipid bilayer lowers the deviation, a size-dependent artifact is still present, indicating scope for improvement.

In the work of Rocklin et al., they solved the finite-size artifact problem by applying a correction that transforms the system from a periodic system with box length *L* into a nonperiodic system of infinite size. This transformation is relatively straightforward for a soluble protein as there is very little ambiguity in defining the nonperiodic system (Figure 1A). However, defining this size-independent nonperiodic system can be nontrivial for a membrane protein (Figure 3A), as it is unclear as to how one should represent the membrane. Given the membrane in a periodic system is, by definition, infinite, there should also be an infinite membrane in the nonperiodic system as well.

**Figure 3:**
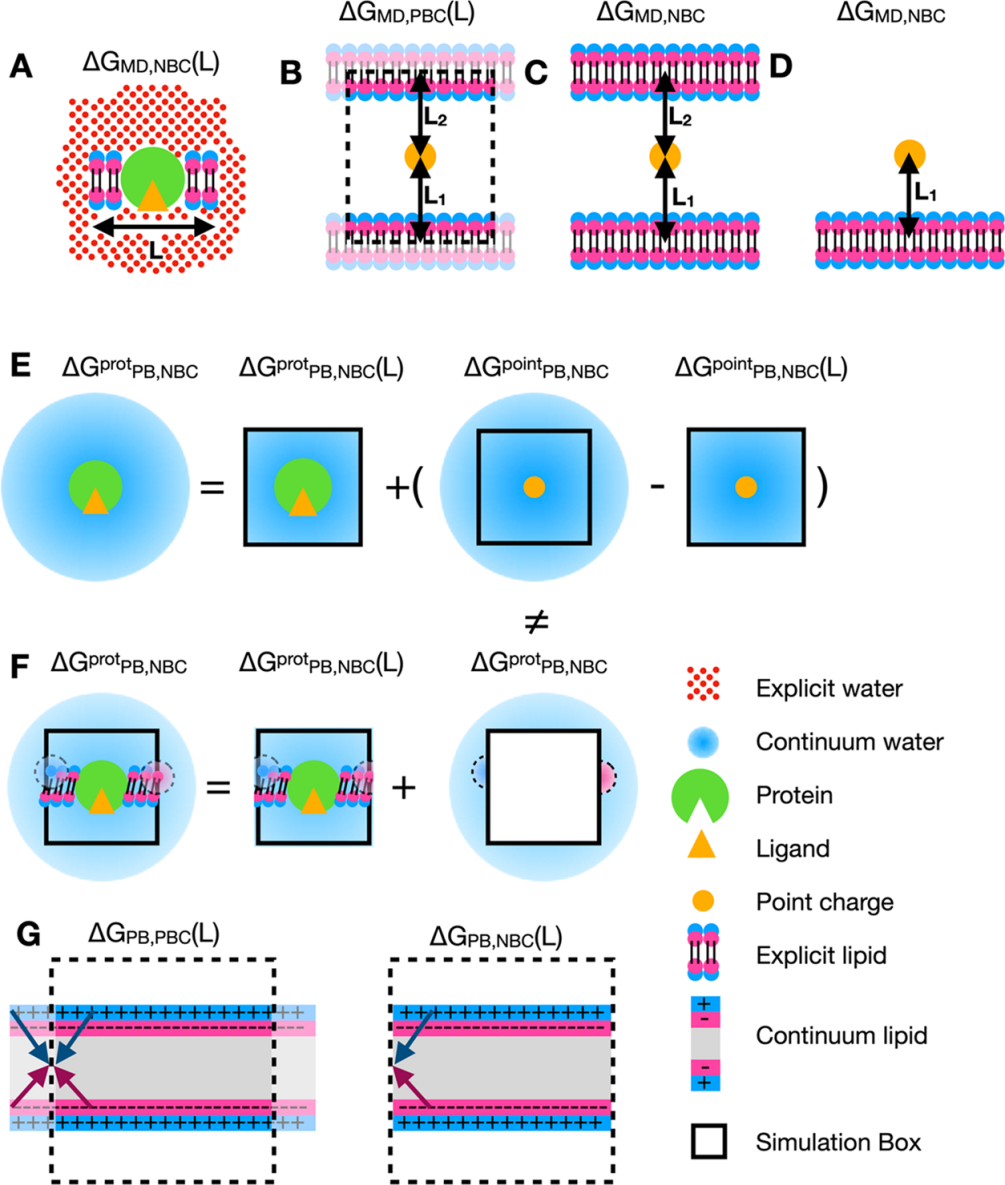
Problems with defining the nonperiodic boundary condition of a lipid membrane system. (A) Defining the nonperiodic boundary condition for a membrane protein system as a membrane patch floating in a macroscopic droplet might not be a good representation. (B) In periodic boundary condition systems, a single membrane could sandwich the ion (orange circle). The nonperiodic boundary condition could be defined as an ion sandwiched by two infinite membranes (C) or the case where only the membrane closest to the ion is preserved (D). (E) The nonperiodic boundary condition for a soluble protein in a continuum solvent can be defined as the sum of the simulation box calculated explicitly with APBS calculations and the space beyond the simulation box calculated analytically, assuming the protein is a naked point charge. (F) The same procedure cannot be performed for membrane proteins as the effect of the lipid extends beyond the simulation box and cannot be approximated as a naked point charge. (G) The lipids outside the box will also affect the lipids in the simulation box, which makes the Poisson–Boltzmann calculation of the simulation box different from the case under periodical boundary conditions.

However, this creates a problem of how to reproduce the position of the net charge with respect to the membrane in the nonperiodic system. In the periodic system, unless the solute is centered exactly at the core of the membrane, the solute is always sandwiched between two membranes, characterized by two distances from both membranes. An extreme case would be the computation of charge annihilation free energy of a single ion in a lipid–water box (Figure 3B). One could argue that two lipid membranes need to be present to reproduce this sandwich effect (Figure 3C). On the other hand, in the periodic system, only one membrane is present in the simulation box, so it might be difficult to map the single-membrane periodic system to the double-membrane nonperiodic system and one should stick with a single membrane in a nonperiodic system (Figure 3D).

The difficulty in defining the nonperiodic condition for lipid bilayer systems poses a challenge during the ΔΔ*G*_RIP_(*L*) calculations. The ΔΔ*G*_RIP_(*L*) corrects for the nonzero average electrostatic potential of the simulation box in nonperiodic conditions. However, no Poisson–Boltzmann solver can compute the electrostatic potential of a macroscopic nonperiodic system. Thus, a key assumption has to be made that the electrostatic potential exerted by a protein would be the same as the electrostatic potential exerted by a naked point charge of the same net charge beyond a certain distance. Thus, the electrostatic potential of a protein system in the nonperiodic condition would be the sum of the electrostatic potential of the protein in a finite-size box and the electrostatic potential of a naked point charge outside this finite-size box (Figure 3E). This assumption holds true for a soluble protein when the protein is at least 1 nm away from the box edge.

It is, however, difficult to argue that this assumption for a soluble protein still holds for a membrane protein. If a single infinite lipid membrane is assumed, lipids will be touching the box edge and the lipid outside the box would exert an electrostatic potential back into the simulation box. This creates a problem both outside and inside the box.

Given that the lipid membrane outside the box is not a homogeneous continuous medium, the original assumption that ΔΔ*G*_RIP_(*L*) + ΔΔ*G*_USV_(*L*) expands the system to a size-independent nonperiodic system no longer holds. Furthermore, lipids touching the boundary will exert an electrostatic potential outside the box that cannot be captured by an analytical solution (Figure 3F).

In both periodic and nonperiodic systems, the lipid on the box edge will experience electrostatic potential effects from lipids outside the box. However, it is difficult to take into account the atoms outside of the simulation box (Figure 3G). For the simplest case of a single membrane in a water box, this boundary effect can be observed in the hydrophobic regions close to the box edge (Figure 4G). Thus, the average electrostatic potential (ESP) computed using a finite simulation box cannot be approximated as the box of the same size sculpted from an infinite membrane system.

**Figure 4:**
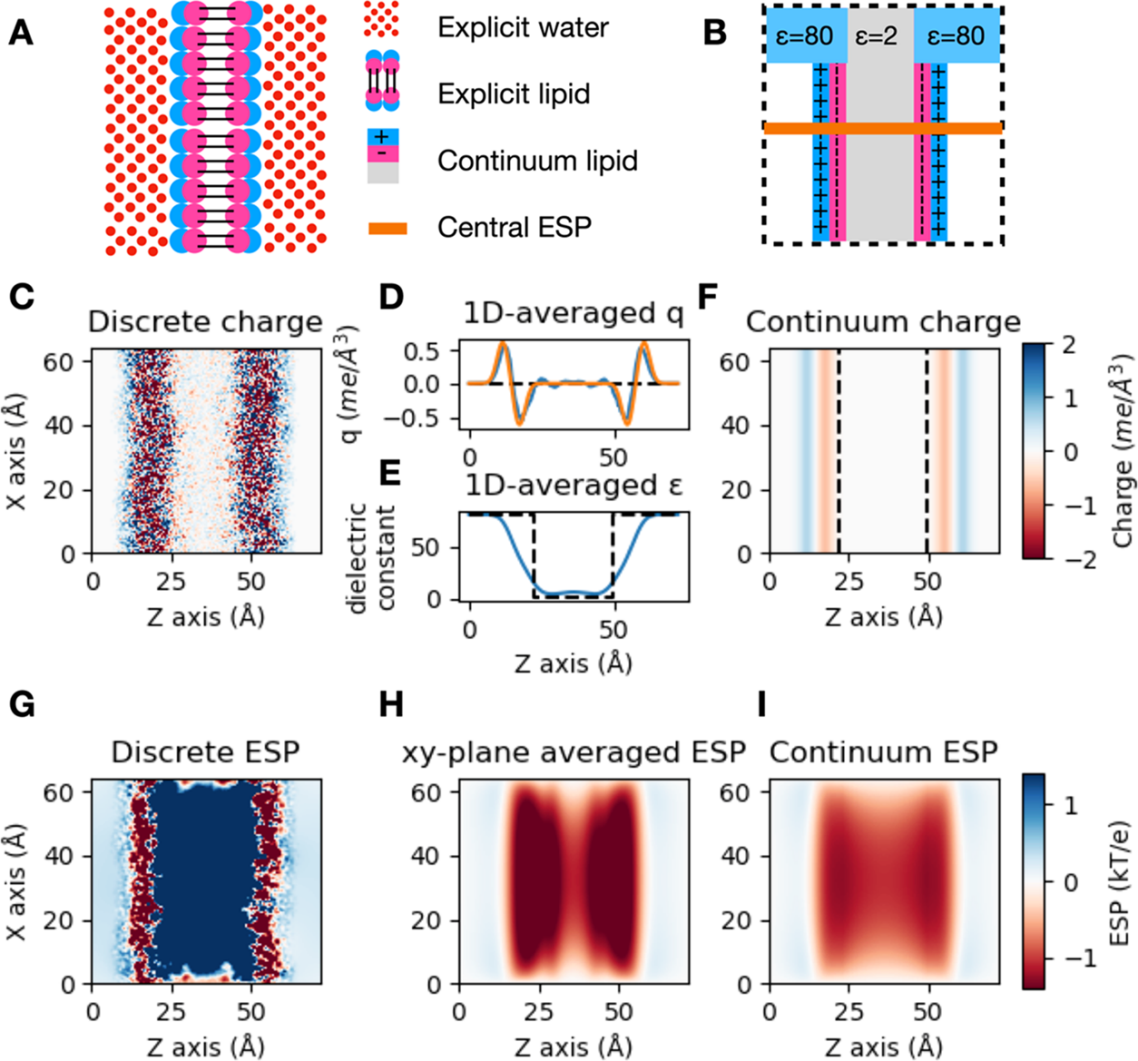
Simple continuum model to represent an explicit lipid. (A) The nonperiodic lipid–water system represented with explicit atoms. (B) The continuum model of the lipid–water system, where the lipid head group is represented by two oppositely charged Gaussian densities and the space is separated into a high dielectric constant zone (water and lipid head groups) and a low dielectric constant zone (hydrophobic core of the lipid). The central ESP is the ESP profile at the center of the lipid along the *z*-axis (orange). (C) The average charge density from APBS calculations with explicit lipid. (D) The average charge density reduced to the *z*-axis (blue) and the fitted charge density with two oppositely charged Gaussian densities (orange). (E) The average dielectric constant reduced to the *z*-axis (blue) and the two-step binary model of high and low dielectric constant regions (dashed black). (F) The separation between high and low dielectric constant regions (dashed black) superimposed on the oppositely charged Gaussian density charge model. (G) The average ESP derived from the APBS calculation with explicit lipid, where a boundary effect can be seen close to the edge. (H) The ESP computed with the average dielectric constant and average charge density from the APBS calculation with explicit lipid. (I) The ESP computed with the continuum lipid model.

One way of mimicking the infinite membrane in the nonperiodic boundary condition is to perform a Poisson–Boltzmann calculation in a box that is much larger than the simulation box and sculpt the simulation box from the result. However, though in theory this would give the average electrostatic potential of the simulation box in nonperiodic conditions, it raises many problems. As is seen in Figure 2E, lipids exhibit large fluctuation between frames (with a standard deviation of 0.7 kT/e) and, thus, a large number of frames need to be considered to obtain a converged value. Furthermore, the large number of lipid atoms that are required to mimic an infinite membrane make the calculation distinctly unattractive for postsimulation treatment.

### Use of a Continuum Model for the Lipid

Given the issues above, we next considered using a continuum model (Figure 4A,B) to replace the explicit lipid as this would make the calculation of a very large lipid feasible and removes the frame-to-frame fluctuation problems. For an infinite lipid membrane, the electrostatic potential would be the same in the plane parallel to the membrane and is a function of the distance to the center of the membrane. This electrostatic potential profile of an infinite lipid membrane could be approximate as the central ESP of a sufficiently large membrane patch along the axis orthogonal to the membrane (Figure 4B).

APBSmem^41^ offers a method of modeling the lipid as two regions of different dielectric constants, where the head group has a dielectric constant of 80 (for the lipid head group and the solvent) and the hydrophobic core has a dielectric constant of 2, as summarized in eq 5 and Figure 4B
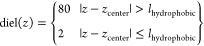
5where diel(*z*) is the dielectric constant of the system as a function of its location on the *z*-axis, *z*_center_ is the center of the lipid membrane, and *l*_hydrophobic_ is the length of the hydrophobic core defined as the distance from the first carbon after glycerol to the end of the aryl tail. Given that the APBS calculation is performed to correct for artifacts arising from molecular mechanics simulations, the dielectric constant of the solvent should match the dielectric constant of the water model (TIP3P) used during the calculation. However, this would mean that the space is segregated into three zones of different dielectric constants, solvent (97 for TIP3P water^42^), lipid head group (80), and lipid hydrophobic core (2). Given that the dielectric constants of the TIP3P water and the lipid head group are quite close, for the simplicity of the calculation, the dielectric constant of the solvent was set to 80 in the following calculations.

Representing the lipid as a hydrophobic slab is insufficient, as is illustrated by large *I*_P_/*L*^3^ (∼1 kT/e) where the partial charge of the lipid plays a significant role during the APBS calculation as well. Previous work^43^ has been done to incorporate the charge effect of the lipid, where the negatively charged phosphate group and the positively charged head group (e.g., choline for phosphatidylcholines) were represented as a pair of ± charge sheets.

A similar continuum lipid model has been constructed to see if a more accurate ΔΔ*G*_RIP_(*L*), which is derived from the average ESP of an infinite lipid membrane, could lower the deviation to the reference charge annihilation free energy. The dielectric constant profile is constructed in the same way using a binary step model (Figure 4E). The charge density of phosphate and choline groups is modeled as Gaussian-shaped charges, as a single sheet of charge might make the calculation sensitive to the box size and grid spacing, as summarized in eqs 6–8
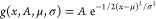
6
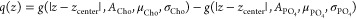
7*q*(*z*) is the charge density as a function of the position on the *z-*axis. The *A*_Cho_, μ_Cho_, σ_Cho_ and *A*_PO_4__, μ_PO_4__, σ_PO_4__ are the magnitude, the center, and the spread of the charge density of the choline group and the phosphate group, respectively. The following constrain on the magnitude and spread has been used to ensure that the sum of the charge density would be zero
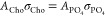
8The parameters of *A*_Cho_, μ_Cho_, σ_Cho_, *A*_PO_4__, μ_PO_4__, and σ_PO_4__ were fitted to a small lipid–water model system (64.26 * 67.47 * 71.67 Å) (Figure 4C). Though there was charge density arising from the small dipole of the aryl chain, they were ignored (Figure 4D). The charge density modeled with two Gaussians and the dielectric constant modeled with a binary step model (Figure 4F) were then used to calculate the ESP (Figure 4I). The ESP computed using the continuum model (Figure 4I) deviated a lot from the average ESP computed using explicit atoms (Figure 4G).

This deviation is not unexpected as the lack of explicit screening will affect the computed electrostatic potential. To compensate for the absence of explicit screening, the use of a coarse-grained Martini model preserves the total charge of the residue but reduces the dielectric constant to 15.^44^ On the other hand, another strategy, Choe et al.,^43^ is to preserve the dielectric constant but scale the charge sheet until the internal potential at the center of the bilayer reaches +300 mV. To confirm that the deviation does indeed come from the lack of explicit screening, the average charge density (Figure 4C) and dielectric constant (Figure 4E) profile from the explicit APBS calculations were used to reconstruct the ESP (Figure 4H), and as expected, it was similar to the result from the continuum model (Figure 4I).

Attempting to reproduce the ESP obtained without explicit screening, the dielectric constant profile was fitted with a logistic function to reproduce the smooth transition from the high dielectric constant to low dielectric constant region, as described by eq 9
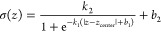
9where *k*_1_, *k*_2_, *b*_1_, and *b*_2_ are fitted to reproduce the dielectric constant profile (Figure 5A), and the fitted parameters are in Table S1.

**Figure 5:**
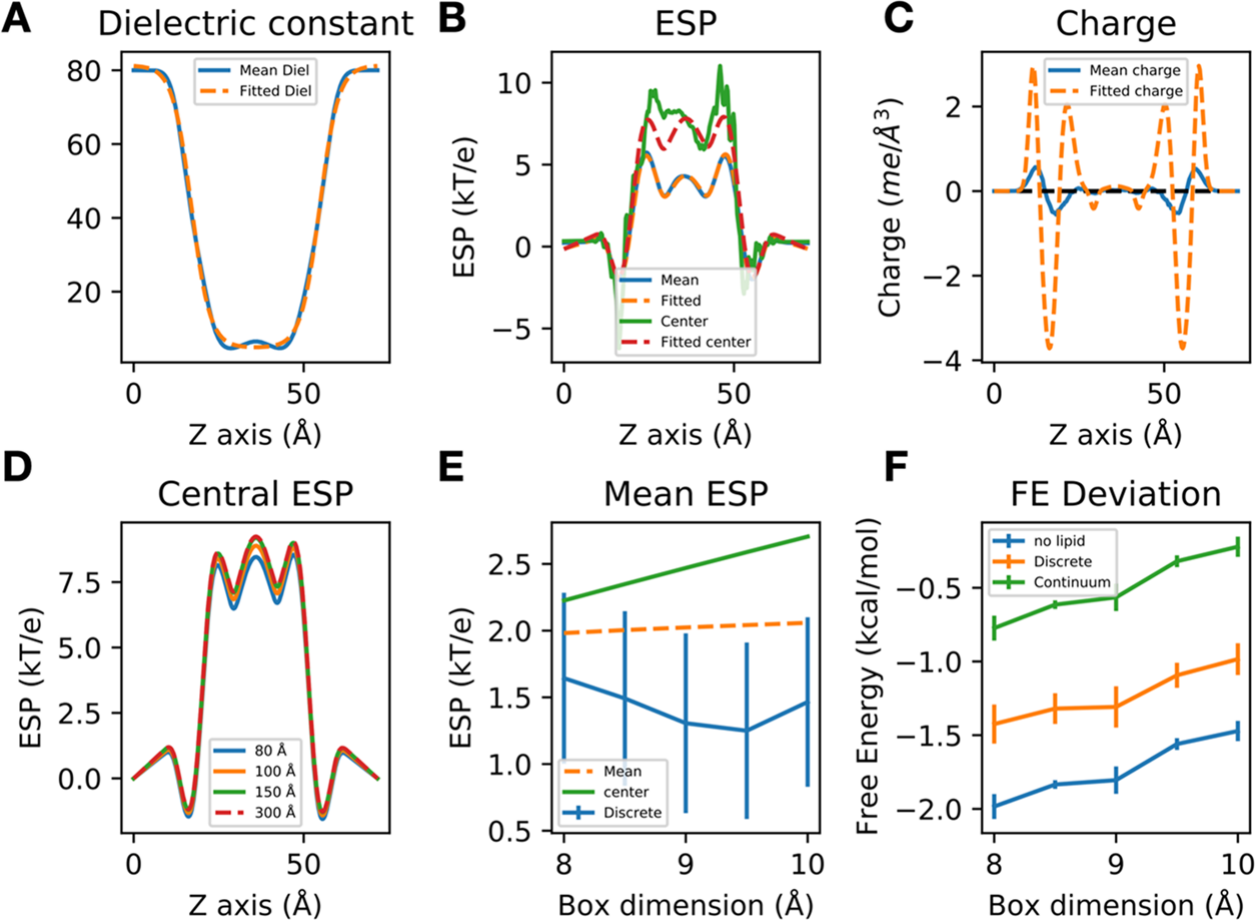
Continuum model reproduces the average ESP and lowers the deviation to the reference free energy. (A) The average dielectric constant profile is fitted with a logistic function. (B) The charge density profile of five Gaussians is fitted (dashed orange line) to reproduce the average ESP computed from APBS calculations with explicit lipid (blue line). The central ESP computed with the continuum model (dashed red) closely matches the result from explicit lipid (green). (C) The fitted charge density bears a similar shape to the average charge density but each Gaussian has a different magnitude. (D) The central ESP is converged when the box dimension reaches 150 Å (green), which is the same as a box dimension of 300 Å (dashed red). (E) The mean ESP computed with the continuum model (orange dashed) is similar to that computed with the explicit lipid (blue). The central ESP with a box dimension of 150 Å gives a different profile (green). (F) The Rocklin correction performed with explicit lipid (orange) would lower the deviation from the reference value compared with not taking the lipid into account (blue), and using the central ESP from the continuum model further lowers the deviation (green).

Five Gaussians were required to reproduce the ESP profile averaged across the *x*–*y*-plane (Figure 5B), and the fitted parameters are given in Table S2. The central ESP profile of the discrete model, though not the direct target of the fitting procedure, matches with that of the continuum model (Figure 5B).

The fitted charge density is written as
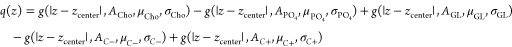
10where the magnitude *A*, center μ, and spread σ of the choline Cho, phosphate PO_4_, ester GL (possibly), and the positive and negative dipoles of the aryl chain *C*+/*C*– were allowed to fit freely. The center of the positive dipole of the aryl chain μ_C+_ is fixed to 0. The new charge density has a similar shape to the original charge density (Figure 5C), where the magnitude is increased to compensate for the lack of explicit screening.

To obtain the ESP profile of an infinite lipid membrane, the size of the membrane patch was expanded in the *xy*-plane until the central ESP was converged (Figure 5D), where a membrane patch with dimensions 150 * 150 Å in the *xy*-plane gives the same central ESP as a membrane patch of 300 * 300 Å.

In terms of calculating ΔΔ*G*_RIP_(*L*), the integrated potential *I* is computed as
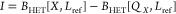
11where *B*_HET_ [*X*,*L*_ref_] is the integrated potential of the simulation box and is defined as
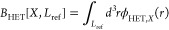
12where ϕ_HET,*X*_ (*r*) is the ESP. *B*_HET_ [*Q_X_*,*L*_ref_] is the reference integrated potential computed with a naked point charge in a box with the dimension of *L*_ref_ and is defined as
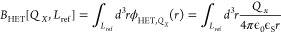
13

Since the lipid has a total charge of 0, *B*_HET_ [*Q_X_*,*L*_ref_] evaluates to 0 in this case.

We transformed the *B*_HET_ [*X*,*L*_ref_] such that it is the ESP at the equivalent position in the *z*-axis from the center of the membrane ϕ_HET,*X*_ (*z*). Thus, *B*_HET_ [*X*,*L*_ref_] is defined as
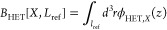
14The ΔΔ*G*_RIP_(L) computed with the center ESP of a large continuum lipid membrane gives very different *I*_P_/*L*^3^ compared with the average ESP of the cubic simulation box with the same *Z* dimension (Figure 5E), and the result from the center ESP lowers the deviation from the reference value (Figure 5F). Note that in terms of computing *I*_P_/*L*^3^ from the simulation box, the continuum lipid model gives a similar value compared with the result computed with explicit atoms for lipids (Figure 5E). Also note the contribution from the ΔΔ*G*_RIP_(*L*) is at a scale of ∼1 kcal/mol. During the lipid APBS calculations, the dielectric constant of the water, for simplicity, was set to 80, instead of 97 for TIP3P water. If the dielectric constant was set to 97 instead of 80, we would expect the ΔΔ*G*_RIP_(*L*) to be 1 * 80/97 ≈ 0.82 kcal/mol. Since the difference of ∼0.18 kcal/mol is much smaller than the force field error, which is usually estimated to be ∼1 kcal/mol, we conclude that setting the dielectric constant of the water to 80 is a reasonable approximation.

### Treatment of Residual Errors

The previous section described our attempts to solve the problem that a Poisson–Boltzmann calculation done with a finite-size simulation box cannot give the same average ESP as the same box in a nonperiodic condition due to the lack of electrostatic interactions from the lipid outside the box (Figure 3G). However, the undersolvation of the ion ΔΔ*G*_USV_(*L*) by the lipid outside the simulation box is still not properly accounted for (see Figure 3F). This can be addressed by deriving a further correction that builds on the Rocklin correction such that it can be applied to the periodic lipid–water system.

The sum of ΔΔ*G*_NET_(*L*) and ΔΔ*G*_USV_(*L*) describes the periodic self-interaction of a naked point charge in a homogeneous medium described by a dielectric constant ϵ_S_ and is defined as
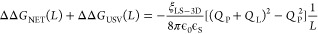
15where ξ_LS-3D_ is the cubic LS (Wigner) integration constant ξ_LS-3D_ ≈ −2.837297;^45^ ϵ_0_ is the permittivity of vacuum, where  evaluates to 138.93545585 kJ nm e^–2^ mol^–1^; ϵ_S_ is the dielectric constant of the solvent, which in this case is TIP3P water ϵ = 97; *Q*_P_ and *Q*_L_ are the total charge of the protein and the ligand, respectively; and *L* is the box dimension.

For a test case of computing the charge annihilation free energy of a chloride ion, the self-interaction in the pure water box can be computed analytically by approximating the water to a homogeneous continuum medium
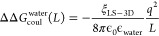
16where *q* is the total charge of the ion and ϵ_water_ is the dielectric constant of the water.

For a single ion in the lipid–water system (Figure 6A), the situation is different as the horizontal interactions parallel to the membrane are through the water medium (ϵ_water_), while all of the off-plane interactions are through a mixture of lipid and water (ϵ_mix_). Thus, the self-interaction term ΔΔ*G*_coul_^lipid-water^(*L*) is defined as
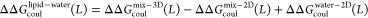
17where ΔΔ*G*_coul_^mix-3D^(*L*) is the self-interaction in the 3D space with a homogeneous medium of ϵ_mix_, ΔΔ*G*_coul_^mix-2D^(*L*) is the self-interaction in the 2D plane, and ΔΔ*G*_coul_^water-2D^(*L*) is the self-interaction in the 2D plane with another homogeneous medium such as ϵ_water_. The terms are defined as
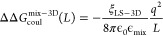
18
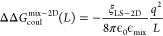
19
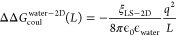
20where ξ_LS-2D_ is the squared LS (Wigner) integration constant ξ_LS-2D_ ≈ −3.90025^45^ and ϵ_mix_ is the dielectric constant of the water–lipid system
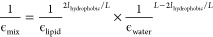
21where *l*_hydrophobic_ is the length of the aryl chain thickness of a single lipid (*l*_hydrophobic_ ≈ 18.87 Å, computed based on the distance between the first atom in the aryl chain to the end of the lipid on 100 ns of equilibrium simulation of a 1-palmitoyl-2-oleoyl-phosphatidylcholine (POPC) bilayer in an NPT ensemble); a factor of 2 is used to account for the bilayer. ϵ_lipid_ is the dielectric constant of the lipid aryl chain (ϵ_lipid_ = 2).^41^

**Figure 6:**
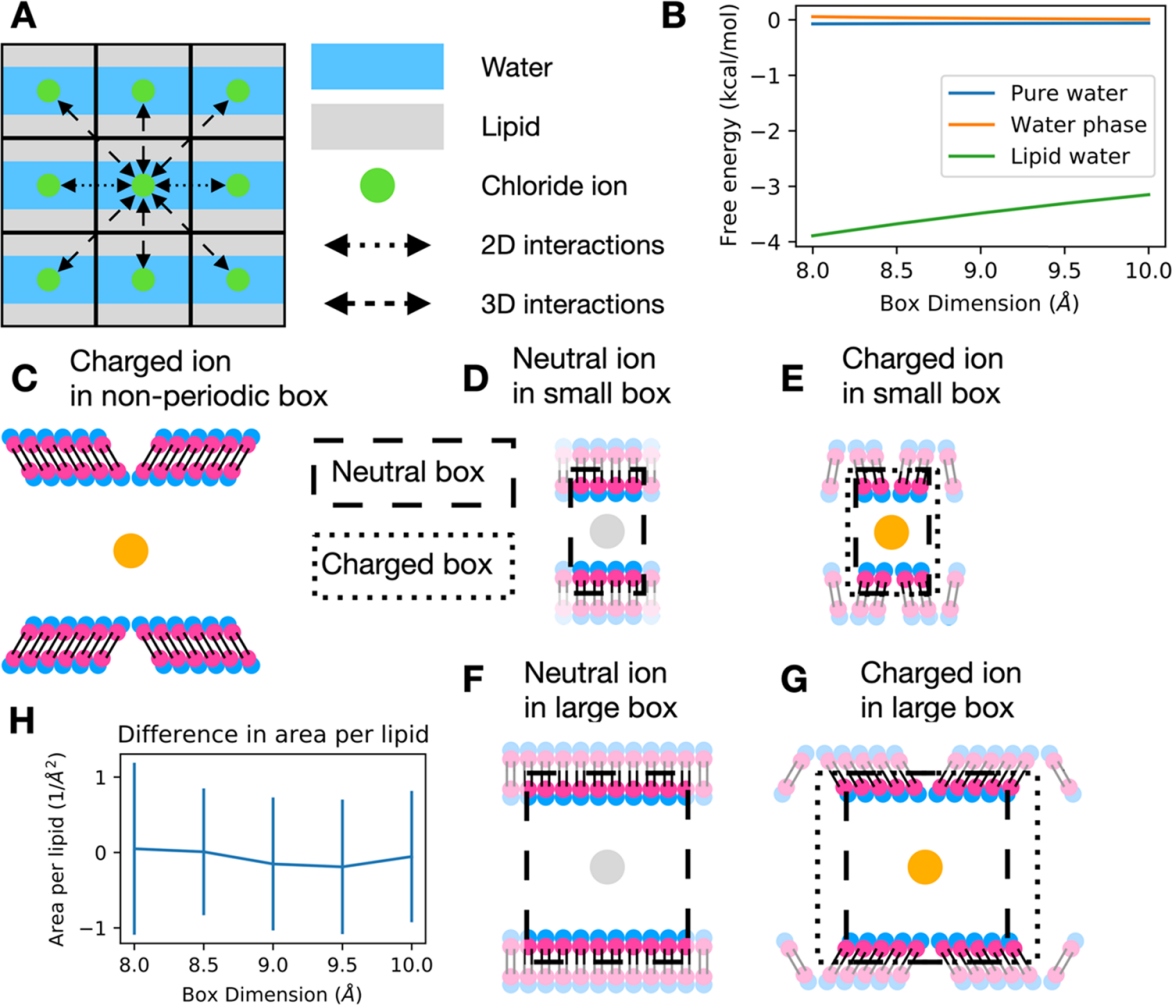
Self-interaction term in a lipid–water system and the discrete solvent effect of the lipid. (A) The plane self-interaction of the ion is through the water phase, whereas the off-plane self-interactions are through the lipid–water mixture. (B) The self-interaction is very small and exhibits very little size dependency when the ion is in a pure water medium (blue) or in the water phase of the lipid–water system (orange). The self-interaction term could be significant for the case where the charged particle is in the lipid phase within a lipid–water system (green). (C) In nonperiodic conditions, the lipid can reorientate in response to the charged ion. In periodic conditions, the lipid will not reorientate when the ion does not harbor charge. (D, F) When the ion is charged, the lipid can reorientate but the extent of the reorientation depends on the size of the box. (E, G) A larger box would permit a larger reorientation and a larger change in the box dimension. (H) The area per lipid as a function of box dimension.

ϵ_water_ is the dielectric constant describing interactions in the *xy*-plane. Since the charge species is an ion in the water phase, ϵ_water_ is the dielectric constant of water and if the charge species is at the center of the membrane (e.g., charged ligand in the membrane protein), ϵ_water_ would be the dielectric constant of the hydrophobic core of the membrane.

To show the effect of this treatment and its influence on the finite-size artifact, the self-interaction terms have been computed using three test cases: monovalent ion in pure water, monovalent ion in the water phase of a lipid–water system, and a hypothetical monovalent ion in the hydrophobic core of the lipid–water system (Figure 6B). Note that for the ion in the lipid–water system, where the 2D plane (i.e., *xy*) self-interaction is through the continuum medium of water (ϵ_water_), the ΔΔ*G*_coul_^lipid-water^(*L*) is very small (<0.1 kcal/mol) but can be quite significant (∼4 kcal/mol) when the net charge is in the hydrophobic core of the lipid bilayer (e.g., a charged ligand in the membrane).

### Discrete Solvent Correction for Lipid

Though the continuum lipid model can reproduce the ESP generated by the explicit lipid in a neutral state, it cannot reproduce the configurational change of the lipid in response to a charged ion. For water, the effect of the charged species on the orientation of the solvent (Figure 1C) is accounted for by ΔΔ*G*_DSC_(*L*). In theory, these effects should still exist for lipids, where the lipid would reorient in response to the charged species in the nonperiodic conditions (Figure 6C). Periodic boundary conditions would be expected to disrupt this lipid reorientation.

This disruption would also be size-dependent. For large boxes, the lipid is not reoriented when the ligand is unchanged (Figure 6F) but would reorient when the ligand is charged (Figure 6G). The reorientation would result in an expansion in the *xy*-plane and a larger area per lipid. For smaller boxes, when the ligand is unchanged, the lipid is still not reoriented (Figure 6D). However, when the ligand is charged, the lipid should reorient but is disrupted by the periodic boundary (Figure 6E). This would result in a smaller increase in the *xy*-plane and a smaller increase in area per lipid. To check if this effect is significant and if it is size-dependent, the difference in area per lipid between the charged and uncharged states has been computed for different box sizes. As shown in Figure 6H, the change of the area per lipid when the ion is charged is very small, suggesting that there is very little reorientation. Furthermore, the effect of the box size on this difference is very small, suggesting that ΔΔ*G*_DSC_(*L*) from lipid reorientation would be negligible.

### Additional Co-Alchemical Ion as Alternative Solution

In the previous sections, we have shown that Rocklin correction could be used to remove the size-dependent artifact during charge annihilation free-energy calculations and, using a continuum model for the lipid bilayer, the deviation from the reference value could be lowered from ∼2 kcal/mol to ∼0.5 kcal/mol for a simple lipid–water system. However, it is unclear as to how such a continuum model for a lipid bilayer would be applied to a protein–lipid system. Another way of solving the size-dependent artifact is to use a co-alchemical ion to maintain the neutrality of the box.

There could be two ways of applying such an alchemical ion. For example, for the charge annihilation of a ligand with a charge of −1, a sodium ion of +1 charge could be annihilated simultaneously or a chloride ion with zero charge could be recharged to a normal chloride ion. For a water system (Figure 7A), both approaches yield the correct answer, where the simultaneous annihilation of chloride and sodium ion yields the sum of the annihilation free energy of a chloride and a sodium ion (Figure 7D). Some deviation can be seen where the coannihilation free energy has some very small size dependency. This deviation could be removed by accounting for the interaction between the sodium and chloride ions, assuming that the water is a continuum medium (Figure 7D).

**Figure 7:**
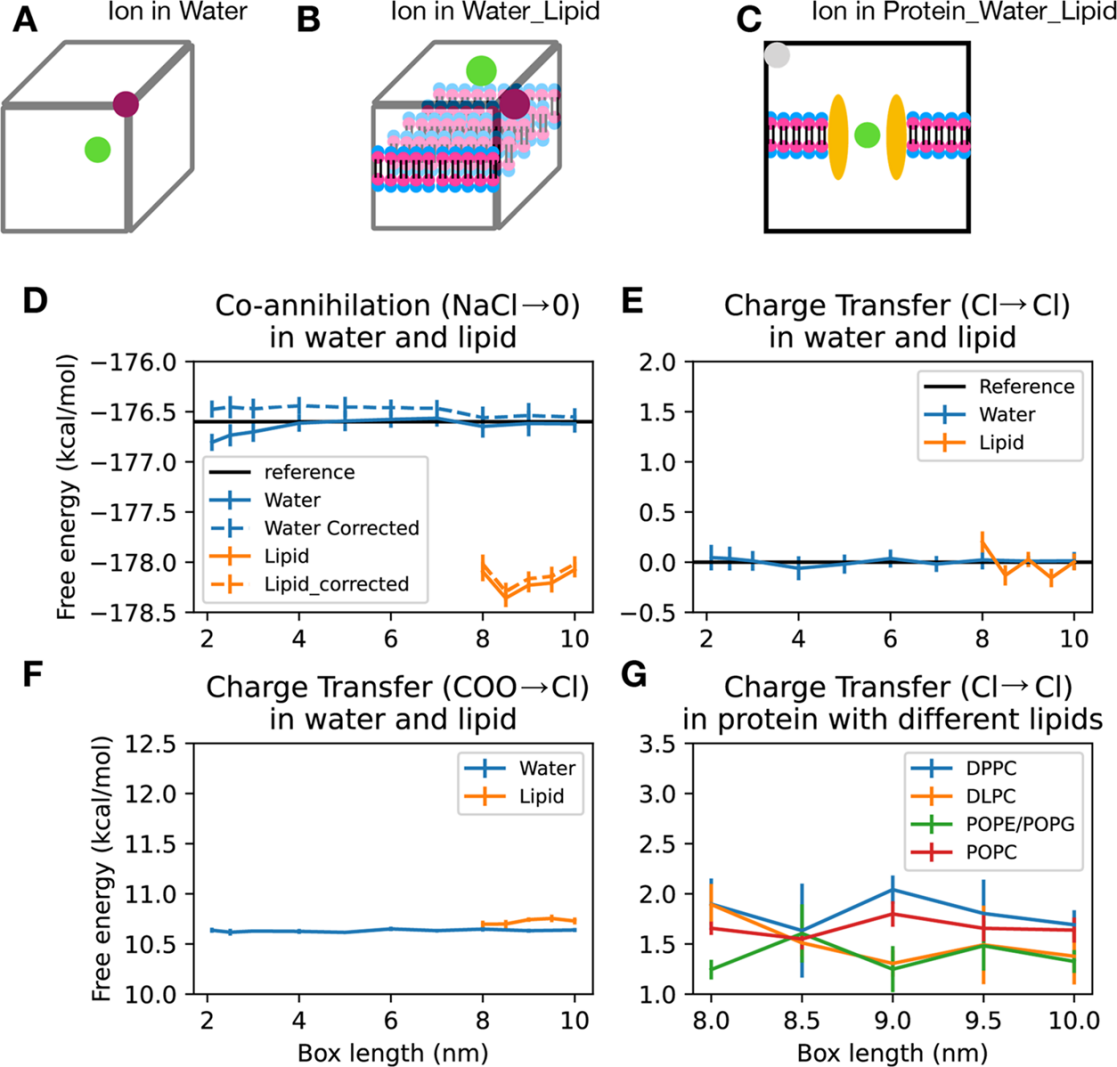
Use of a co-alchemical ion to maintain charge neutrality. (A) The test system for the coannihilation of NaCl or charge transfer from the chloride ion or formic acid to chloride ion, where one molecule is put at the center of the box and the other at the edge of the box. (B) The test case for the lipid–water system, where one molecule is put at the center of the box and the other molecule at the same *xy*-plane, is at the edge. (C) The test case where protein is embedded in the lipid and the chloride ion is restrained in the center of the protein, where the charge is transferred from the chloride (green) in the protein (brown) to another chloride at the corner of the box (gray). (D) The coannihilation free energies of NaCl in water (blue) and the lipid–water system (orange) are different. (E) The free energy of charge transfer from chloride to chloride in both pure water and lipid membrane systems. (F) The charge transfer from formic acid to chloride ion. (G) The free energy of charge transfer of the chloride ion in a membrane protein to a chloride ion in solvent in different lipids.

Another option is to charge another ion to keep the total charge neutral. To account for the charge annihilation of a chloride ion, another neutral chloride ion will be charged. The charge transfer from one chloride ion to another chloride ion yields a zero free-energy difference, regardless of the presence of lipid (Figure 7E). Since the charge transfer between chloride ions will always give zero, formic acid is used as a test case, where the charge-transfer free energy is constant across different box sizes (Figure 7F).

The situation is, however, different for lipid–water systems (Figure 7B), where the simultaneous annihilation of chloride and sodium ions yields a free energy of ∼1.5 kcal/mol lower than the case for a pure water system (Figure 7D). Many factors could give rise to this deviation. The ions could have interactions with the lipid bilayer, which are absent in a pure water system. The lipid bilayer could disrupt the solvation shell around the ion in a different sense compared with the ion in a pure water system. Finally, the self-interaction free energy of the ions across the periodic boundary condition could be different due to the low dielectric constant of the lipid acryl tail. Among these three factors, the final factor is the only one that we would consider here. Given that an analytical solution cannot be derived to describe the interactions between the sodium and chloride ions across the periodic boundary, we attempted to remove this artifact numerically. The lattice-sum energy of the system{Rocklin, 2013 #7126} can be described as
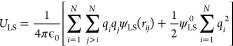
22where the system has *N* charged particles, where each particle has charge *q*_*i*_ at location *r*_*i*_ (*r*_*ij*_ = *r*_*j*_ – *r*_*i*_). ψ_LS_ is the lattice-sum influence function, which is the electric potential generated by a unit point charge at the origin multiplied by 4πϵ_0_, and ψ_LS_^0^ is the Wigner self-term constant (difference between ψ_LS_ and *r*^–1^ in the limit of infinitesimal distances). For a cubic box of edge *L* in a homogeneous medium with a dielectric constant of ϵ_S_, the ψ_LS_ and ψ_LS_^0^ are defined as
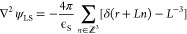
23
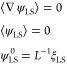
where δ is the three-dimensional Dirac delta function. For the lipid–water system, where the dielectric constant is different for plane interactions and off-plane interaction (Figure 6A), ψ_LS_ is defined as
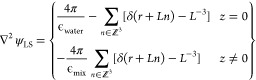
24where ϵ_water_ is the dielectric constant of water and ϵ_mix_ is the dielectric constant of the lipid–water system. *z* is the distance to the center of the water phase and describes the interactions going purely through the water phase in the lipid–water system.

To obtain this lattice-sum energy numerically, three PME calculations were performed
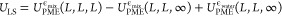
25where *U*_PME_^ϵ_mix_^ (*L*, *L*, *L*) is the lattice-sum energy of the system in a continuous medium of ϵ_mix_, *U*_PME_^ϵ_mix_^ (*L*, *L*, ∞) is the lattice-sum energy of the system in a continuous medium of ϵ_mix_ with an infinite *z*-axis dimension (10,000 nm as employed in the calculation), and *U*_PME_^ϵ_water_^ (*L*, *L*, ∞) is the lattice-sum energy of the system in a continuous medium of ϵ_water_ with an infinite *z*-axis dimension. The lattice-sum energy removes a very small amount of the deviation but the majority persists. Thus, we conclude that the deviation could not be corrected easily and further considerations are required if the simultaneous annihilation method were to be used in lipid membrane systems.

The charge-transfer method, on the other hand, still gives a very small amount of deviation in the membrane system compared with the pure water system. This deviation, however, is negligible (∼0.1 kcal/mol) and relatively size-independent.

### Co-Alchemical Ion in the Case of a Membrane Protein

Although we have shown above that the charge-transfer method using a co-alchemical ion can derive the free-energy difference in a size-independent manner in an idealized system, it remains to be seen as to how it would behave in the real world, where the ligand is bound to a protein embedded in a membrane. We therefore constructed a system with the 14-stranded outer-membrane porin OmpG^46^ embedded in a POPC membrane. The ion to be annihilated is situated at the core of the protein at the same level as the core of the hydrophobic region of the lipid (Figure 7C). Given that the chloride ion in the protein is in an environment that is different from the solvent, the charge transfer from one chloride ion to another chloride ion will not be the same as the case when both of them are in solvent (Figure 7E). As shown in Figure 6B, the self-interactions across the hydrophobic region of the lipid would give a significant self-interaction energy that would be expected to be size-dependent; this problem should be greatly reduced when the ligand is in the protein. Our calculations show that for the model protein system, this effect of the box dimension on the self-interaction term is smaller than the error and can be safely ignored (Figure 7G). Furthermore, the result is invariant with respect to the lipid saturation level (saturated DPPC, half-saturated POPC) or thickness (short DLPC/long DPPC) or when the lipid is charged (POPE POPG mixture) showing that the method is robust.

## Discussion

In this work, we have shown that the Rocklin correction can correct for the finite-size artifact for the soluble system but will exhibit a large deviation in the lipid–water system. By incorporating a continuum lipid model via the Poisson–Boltzmann calculation, we were able to greatly reduce the error. Although this gives a reasonable improvement, this approach remains rather limited as the continuum lipid model could only be derived for a pure lipid bilayer and it is unclear as to how to generalize this to all lipid–protein systems. Instead, we propose to avoid this problem altogether and advocate the use of the co-alchemical ion approach, where the charge of the charged ligand is transferred to the co-alchemical ion. However, it is also the case that this approach gives rise to a small systematic error when the membrane is present. Note that in the case where a large enough bulk solvent volume can be included, then one might use the same volume to couple another copy of the ligand, thus reverting to the double-system single-box approach {Macchiagodena, 2021 #10371}, but there are practical implications that can make this approach less trivial than a simple co-alchemical ion treatment.

Our results here shed some light on the apparent inconsistencies in the literature, where many people have strongly advocated the Rocklin correction and the necessity of correcting for the finite-size artifact. Others have shown that ignoring the size-dependent artifact can still give results that give a good correlation with the experimental data.^47^ Similarly, there are studies that have shown that the Rocklin correction has very little effect on the RBFE calculations.^48^ This is likely best explained by the fact that the Rocklin correction mainly corrects for the long-range effects of the electrostatic interactions. The ΔΔ*G*_DSC_(*L*), which is usually the largest component in Rocklin correction, depends on the proportion of the box that is filled with water. For RBFE calculations, this proportion is unlikely to change, which means the ΔΔ*G*_DSC_(*L*) is very similar for different ligands tested in RBFE. Though the specific ligand–protein interaction would give rise to a different ΔΔ*G*_RIP_(*L*), different ligands would give a very similar average electrostatic potential and ΔΔ*G*_RIP_(*L*) is usually very small unless lipids are involved.

One way of avoiding the finite-size artifact would be to ensure the neutrality of the simulation box. For the computation of ligand binding free energy, an alternative would be path sampling, where the free energy of pulling the ligand out of the binding pocket is taken as the binding free energy. However, it has been observed that the binding free energy computed with path sampling did not converge to the same end result as those done alchemically and corrected with the Rocklin correction.^49^ However, one study has also shown that they can give similar results.^50^ One explanation for this discrepancy would be that the self-interaction term would be size-dependent when the species of opposite charge cannot sample the same configurational space. For example, in Figure 6B, we have shown that the self-interaction energy of an ion in the water phase of the lipid–water system can be quite different compared with the same ion in the hydrophobic core. Thus, in the case where the charge species cannot sample the whole configurational space, such as when the ligand is restrained to the protein during path sampling, the self-interaction energy of the charged ligand cannot be compensated by an ion of opposite charge in the water phase. Thus, the self-interaction energy of the ligand will be size-dependent and could give different results when the simulation box has different sizes. This difference in the self-interaction energy could also explain the conformational dependence on the box size,^51^ where smaller boxes would favor the conformation that minimizes the self-interaction energy.

## Conclusions

To solve the finite-size effect, we have shown that the Rocklin correction, the co-alchemical ion with charge transfer, and coannihilation of the charge would all work for a soluble protein system. For systems with lipid bilayers, however, only the co-alchemical ion using charge transfer will avoid the finite-size effects, and thus for these kinds of calculations, this is the recommended approach.

## Methods

### Construction of Water Box

To investigate the charge annihilation free energy, empty boxes were constructed, where the box dimension, *L*, was 2.1, 2.5, 3, 4, 5, 6, 7, 8, 9, and 10 nm. For the single-ion charge annihilation free-energy calculations, the ion was placed at the center of the box and the box was solvated with the gmx solvate tools.^52^ For the charge transfer or coannihilation calculations, the chloride ion was position-restrained at the center of the box with a restraint strength of 1000 kJ/(mol nm^2^) at the *x-*, *y-,* and *z*-axes. The sodium ion, chloride ion, or formic acid was position-restrained to the edge of the box (0, 0, 0) at the same strength.

### Construction of the Lipid–Water Box

The lipid–water box was constructed using a pre-equilibrated POPC system obtained from the slipid website (http://www.fos.su.se/~sasha/SLipids/Downloads.html, accessed 09/12/2021).^53^ The lipid was replicated in the *x-* and *y*-dimensions with MDAnalysis^54^ and was trimmed to the desired dimension, *L*, as required (i.e., 8, 8.5, 9, 9.5, 10 nm). The box was solvated with water and equilibrated for 100 ns under isobaric and isothermal ensemble (NPT) conditions. For the charge annihilation of a single ion, the ion was placed at the center of the box and was position-restrained on the *z*-axis with a restraint of 1000 kJ/(mol nm^2^). For the charge transfer or coannihilation calculations, the chloride ion was placed at the center of the box (*L*/2, *L*/2, *L*/2) and the sodium ion, chloride ion, or formic acid was placed at the edge at the same *z*-axis (0, 0, *L*/2) and position-restrained in all (*x*, *y*, and *z*) axes. To ensure the ion remains in the middle of the water phase (in the middle of the box), the equilibrated box was centered with respect to the water phase.

### Construction of the Protein–Lipid–Water Box

The open form of OmpG (PDB: 2IWV)^46^ was embedded in a POPC bilayer using a self-assembly protocol, as described previously by us.^55^ Both protein and lipid were described using the Martini 3 force field.^44^ The lipids were trimmed to the desired box size and equilibrated for 20 ns with the “New-RF” parameters.^56^ The system was then converted to an atomistic representation with cg2at^57^ and then further equilibrated for 100 ns. The ion to be annihilated was position-restrained to the center of the protein (i.e within the barrel), where the reference *z-*axis coordinate is the center of the membrane defined as the mean of the phosphate groups of the lipids. The coordinate in the *x*–*y*-plane was chosen such that it is the furthest from any atoms in the protein. To ensure that the same result is obtained in different box sizes, the protein was position-restrained to the center of the box using the same reference coordinate from the crystal structure. The co-alchemical ion where the charge is created was restrained to the corner of the box [0, 0, 0]. All positional restraints were set to 1000 kJ/(mol nm^2^).

### Simulation Setup

Simulations were run with the GROMACS 2020 MD engine.^52^ The force field for lipids was slipid,^53^ and the TIP3P water model was used for water. Formic acid was parameterized with GAFF2.^58^ The electrostatic interactions and Lennard-Jones interaction were computed with particle mesh Ewald (PME).^5^ The pme-order was set to 6, the Fourier spacing was set to 0.1, and the ewald-rtol was set to 1 × 10^–6^ for electrostatic interactions and 1 × 10^–3^ for Lennard-Jones interactions. The direct space cutoff was set to 1 nm. The H-bond length was constrained by LINCS^59^ with lincs-iter and lincs-order set to 2 and 6, respectively. The time step of integration was 2 fs. The alchemical transformation was done via 11 steps with a delta of 0.1. The charge of the chloride, sodium, or formic acid was scaled linearly from the full charge to zero or vice versa.

The system was energy-minimized before a 100 ps NPT equilibration with Langevin dynamics at 298.15 K,^60^ and the pressure was restrained to 1 bar with the Berendsen barostat.^61^ Production runs employed replica exchange, and exchanges were performed every 1000 steps and used the Parrinello–Rahman barostat.^62^ For the calculations involving only sodium and chloride ions, production runs were 1 ns and were repeated 30 times. For the calculations involving formic acid, five repeats of 30 ns production runs were performed. The free-energy estimate was done with MBAR^63^ and alchemlyb.^64^

### Poisson–Boltzmann Calculations

APBS 1.5^65^ was used to numerically solve Poisson–Boltzmann equations. A POPC water box with a dimension of (64.26 * 67.47 * 71.67 Å) was simulated in the NVT ensemble for 100 ns, where 100 snapshots were taken to compute the electrostatic potential profile. The APBS input files were modeled on the Rocklin correction.^11^

For the computation of the continuum model, the input files (charge density, dielectric constant mesh) were prepared with rocklinc (https://github.com/bigginlab/rocklinc, accessed 11/11/2021) and all analysis codes are included in the same depository.

## Supporting Information Available

Fitted parameters used for the dielectric constant in eq 9 (Table S1) and fitted parameters for the charge density used in eq 10 (Table S2) (PDF)
