# Supplementary material for: Correction Schemes for Absolute Binding Free Energies Involving Lipid Bilayers
Source: J Chem Theory Comput. 2022 Mar 22;18(4):2657–72. doi: 10.1021/acs.jctc.1c01251 (PMC9082507; doi:10.1021/acs.jctc.1c01251)
Supplement: Supplementary file 1 — ct1c01251_si_001.pdf [file ct1c01251_si_001.pdf]

SUPPLEMENTARY INFORMATION

Correction schemes for absolute binding free energies involving lipid bilayers.

Zhiyi Wu* and Philip C. Biggin*.

Department of Biochemistry, South Parks Road, Oxford. OX1 3QU. UK.

*Email: Zhiyi.wu@bioch.ox.ac.uk and Philip.biggin@bioch.ox.ac.uk

Table S1. Parameters for the dielectric constant

**k_1(Å^{-1})**	**b_1 (Å)**
0.37	-18.87
**k_2**	**b_2**
76.49	4.83

Table S2. Parameters for the charge density

	Magnitude (e/Å^3)	Centre (Å)	Spread (Å)
**Cho**	3.30E-03	24.20	1.28
**PO_4**	5.70E-03	18.60	2.41
**GL**	4.18E-03	16.07	2.47
**C-**	4.25E-04	6.60	0.90
**C+**	1.17E-04	0*	2.95

*not optimized.
